# Focus on podocytes: diabetic kidney disease and renal fibrosis — a global bibliometric analysis (2000–2024)

**DOI:** 10.3389/fphar.2024.1454586

**Published:** 2024-11-15

**Authors:** Dong-Yang An, Jun Tan, Yan-Dan Lu, Ze-Huai Wen, Yi-Ni Bao, Zhou-Hui Yao, Zi-Yan Chen, Ping-Ping Wang, Wei Zhou, Qiao Yang, Min Hao

**Affiliations:** ^1^ School of Pharmaceutical Sciences, The First Affiliated Hospital of Zhejiang Chinese Medical University, Hangzhou, China; ^2^ School of Pharmacy, Zhejiang Chinese Medical University, Hangzhou, China; ^3^ The First Affiliated Hospital of Guangzhou University of Chinese Medicine, Guangzhou, China; ^4^ Second Clinical College, Guangzhou University of Chinese Medicine, Guangzhou, China; ^5^ Research Center for Clinical Application of Chinese Medicine Classics, Guangdong Provincial Hospital of Chinese Medicine, Guangzhou, China; ^6^ Department of Nephrology, The Children’s Hospital, Zhejiang University School of Medicine, National Clinical Research Center for Child Health, Hangzhou, China

**Keywords:** podocytes, DKD, renal fibrosis, Poria cocos, holistic integrated medicine

## Abstract

**Background:**

Diabetic kidney disease (DKD) is a common pathway to End-stage renal disease (ESRD). Podocytes are crucial due to their dual barrier functions in kidney diseases. Their role in renal fibrosis and DKD regulatory mechanisms is increasingly studied. However, bibliometric research in this field has not been explored.

**Methods:**

1,250 publications from Jan. 1, 2000, to Feb. 16, 2024, were retrieved from the WoSCC database and analyzed by the Web of Science results analysis tool, VOSviewer, and CiteSpace.

**Results:**

Our scrutiny reveals that authors Liu Youhua, Fogo Agnes B, and Zhao Yingyong have made substantial contributions to this domain. Notably, “Kidney International” has the highest volume of publications in this area. Furthermore, our analysis identifies ten co-citation clusters: DKD, IncRNA, reactive oxygen species, glomerulosclerosis, Poria cocos, glomerular diseases, fibroblasts, connective tissue growth factor, coagulation, and Wnt. Recent research accentuates keywords such as autophagy, TRPC6, ERS, epigenetics, and NLRP3 inflammasome as frequently occurring terms in this field. The prevailing research hotspot keywords include autophagy, biomarker, and exosomes.

**Conclusion:**

Through the utilization of bibliometric tools and knowledge graph analysis, we have undertaken a comprehensive review of the intricate nexus between podocytes in DKD and renal fibrosis. This study imparts valuable insights to scholars regarding the dynamic evolution of this association and delineates prospective research avenues in this pivotal realm.

## Graphical Abstract

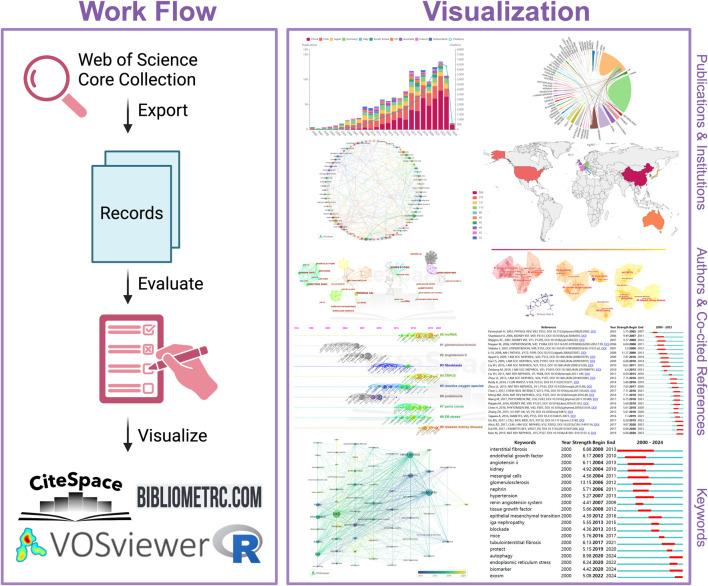

## Highlights

• This is the first bibliometric analysis of 1,250 articles on podocytes, DKD, and renal fibrosis (2000–2024).
• TCM active compound Poricoic acid A (from Poria cocos) can reduce blood glucose and relieve DKD.
• Current hotspots: autophagy, exosomes, AMPK, Wnt/β-Catenin, MALAT1, biomarkers, epigenetics, and epigenomics.
• The research on podocytes, the progression of DKD, and renal fibrosis has seen an explosion in the past decade.
• Holistic integrated medicine (HIM) is a prospective DKD and Renal fibrosis direction.

## 1 Introduction

Globally, Diabetic kidney disease (DKD) (Thomas et al., 2015; KDOQI, 2007) stands as the primary cause of chronic kidney disease (CKD) and end-stage kidney disease (ESKD), accounting for 50% of all cases (Mohandes et al., 2023; Pereira et al., 2022). DKD is characterized by a gradual and progressive decline in kidney function (Calle and Hotter, 2020), culminating in renal fibrosis and eventual organ failure (Ketteler et al., 2018). Renal fibrosis is a common pathological consequence of DKD (Humphreys, 2018). In the glomerulus, capillary lumens are generally observed to be extended, the basement membrane is thickened, the extracellular matrix (ECM) is expanded, podocyte injury is present, and fibrosis is detected. Podocytes are highly specialized epithelial cells, also known as “octopus-like” highly specialized cells in the glomerulus. As part of the kidney filter (Liu et al., 2022; Rosenberg and Kopp, 2017), there is a key component of the glomerular filtration barrier which plays an indispensable role in maintaining the structure and function of the glomerulus. These cells are uniquely positioned at the outer layer of the glomerular capillaries which are critical in preventing the leakage of proteins into the urine. Podocytes extend into the basement membrane via primary, secondary, and tertiary foot processes, maintaining structural stability through an actin cytoskeleton. The approximately ∼200-nanometer-wide space between adjacent podocytes is bridged by the slit diaphragm, a structure that not only acts as an ∼60 kDa size-selective filter for both molecular size and electric charge. To prevent the passage of large molecules but also exhibits charge selectivity, tending to repel negatively charged macromolecules such as plasma proteins, thus ensuring the effectiveness of a dual size and charge-selective filtration mechanism. In cases of disease or injury, podocytes may fail to effectively compensate for physiological stresses such as circumferential stress and shear forces, leading to podocyte loss or increased mechanical load on the remaining podocytes. This can result in ECM deposition and further podocyte loss, accelerating the progression of kidney disease. Podocytes not only maintain the integrity of the glomerular filtration barrier but also play a crucial role in the regulatory mechanisms of renal fibrosis and DKD (Kopp et al., 2020).

Emerging evidence suggests that podocyte dysfunction and loss contribute significantly to the development and progression of renal fibrosis in DKD (Rosenberg and Kopp, 2017). Among the intricate interplay of various cellular and molecular mechanisms underlying DKD pathogenesis, the role of podocytes and their involvement in renal fibrosis has garnered substantial attention in recent years (Jiang et al., 2022). Podocytopathies (Rosenberg and Kopp, 2017; Wiggins, 2007) are kidney diseases that cause direct or indirect injury to the podocytes. Podocytopathies can lead to a decrease in the number of renal units and increase the risk of CKD (Drawz and Rahman, 2015). New evidence shows that podocyte dysfunction and loss have a significant impact on the occurrence and progress of renal fibrosis in DKD (Roccatello et al., 2023). Their injuries resulted in CKD and high risk (Johansen et al., 2023; System USRD, 2022). This condition has a global impact, with an estimated burden of 10% or greater (GBD Chronic Kidney Disease Collaboration, 2020; Murray and Lopez, 2013). While therapeutic interventions can significantly delay the progression of CKD, the long-term outlook remains guarded (Levin et al., 2017; Lv and Zhang, 2019). In academic inquiry, bibliometrics emerges as an indispensable analytical tool, wielding mathematical and statistical techniques to systematically scrutinize the corpus of scholarly literature and various forms of media.

This approach serves as a linchpin in the qualitative and quantitative evaluation of academic domains, encompassing examinations of countries or regions, academic institutions, authors, co-cited authors, journals, references, and keywords. Furthermore, bibliometrics demonstrates its capability to delineate and forecast pivotal research themes and emergent trends within specific disciplinary contexts (Zhang et al., 2023).

Despite the substantial knowledge accumulated in renal fibrosis associated with DKD, the intricate molecular mechanisms underlying podocyte injury and their implications within the context of renal fibrosis remain elusive. In the present study, we utilize bibliometric methodologies to illuminate the landscape of podocyte-related research within the domain of renal fibrosis in kidney disease, with a particular emphasis on the trends in this progressing field of inquiry.

## 2 Methods

### 2.1 Data acquisition and search strategy

Web of Science, renowned for its status as a preeminent and all-encompassing database platform, stands as a paragon of authority and academic excellence, housing an extensive array of scholarly journals. It currently reigns as the most frequently employed database for the purpose of bibliometric analysis (Zhang et al., 2023; Zhang et al., 2022). Consequently, we leveraged the Web of Science Core Collection (WoSCC) as the primary reservoir of data for our research endeavor.

In our quest for meticulousness and precision in data retrieval, we opted for the SCI-Expanded citation index. To ensure the inclusiveness and fidelity of our dataset, we executed a search query encompassing the keywords: “[(TS = (renal fibrosis)) OR TS = (kidney fibrosis)] AND TS = (podocyte*) AND Document types = (Article OR Review Article OR Early Access) AND Language = (English)”. This search was conducted over a defined temporal scope spanning from 1 January 2000 to 16 February 2024.

Moreover, it is imperative to note that all pertinent bibliographic information, publication year, title, authors, countries, institutional affiliations, abstracts, keywords, and publishing journals, was exported as a plain text file from the WoSCC database.

### 2.2 Bibliometric study and visual representation

Bibliometrics, constituting an autonomous scholarly discipline, furnishes quantitative methodologies essential for thoroughly scrutinizing and investigating extant literature within a specific academic domain (Zhang et al., 2023; Meng et al., 2022). Through this analytical process, a wealth of granular information encompassing authorship, keywords, journal outlets, geographical origins, institutional affiliations, references, and the like can be meticulously gleaned.

The application of visualization techniques serves as a potent tool in elucidating the inherent interrelationships among this multifaceted information landscape. This encompasses revelations such as distinct authors converging on a common research trajectory, divergent research emphases emanating from disparate institutions, novel theoretical paradigms emerging within established academic bastions, and more.

We conducted a comprehensive analysis of the articles from the perspectives of publication trends, citations, countries/regions, institutions, authors, journals, references, keywords, cooperative relationships, etc., using VOSviewer (Song et al., 2022) and CiteSpace (Sabe et al., 2022) software.

Furthermore, within this analysis, Taiwan has been categorized under the rubric of the People’s Republic of China; similarly, England, Scotland, North Ireland, and Wales have been collectively classified under the aegis of the United Kingdom.

## 3 Results

### 3.1 Global publication and citation trends

In the time spanning from 2000 to 2024, our study meticulously gathered a corpus of 1,250 scholarly articles from the esteemed WoSCC. This dataset encompassed 951 research articles, 266 reviews, and 18 early-access articles, all uniformly published in English. Notably, these 1,250 contributions, citing 29,902 articles pivotal to our investigation, bore the intellectual imprint of 6,342 distinct authors from 1,462 academic institutions distributed across 62 countries/regions, emanating from 366 journals.

**FIGURE 1:**
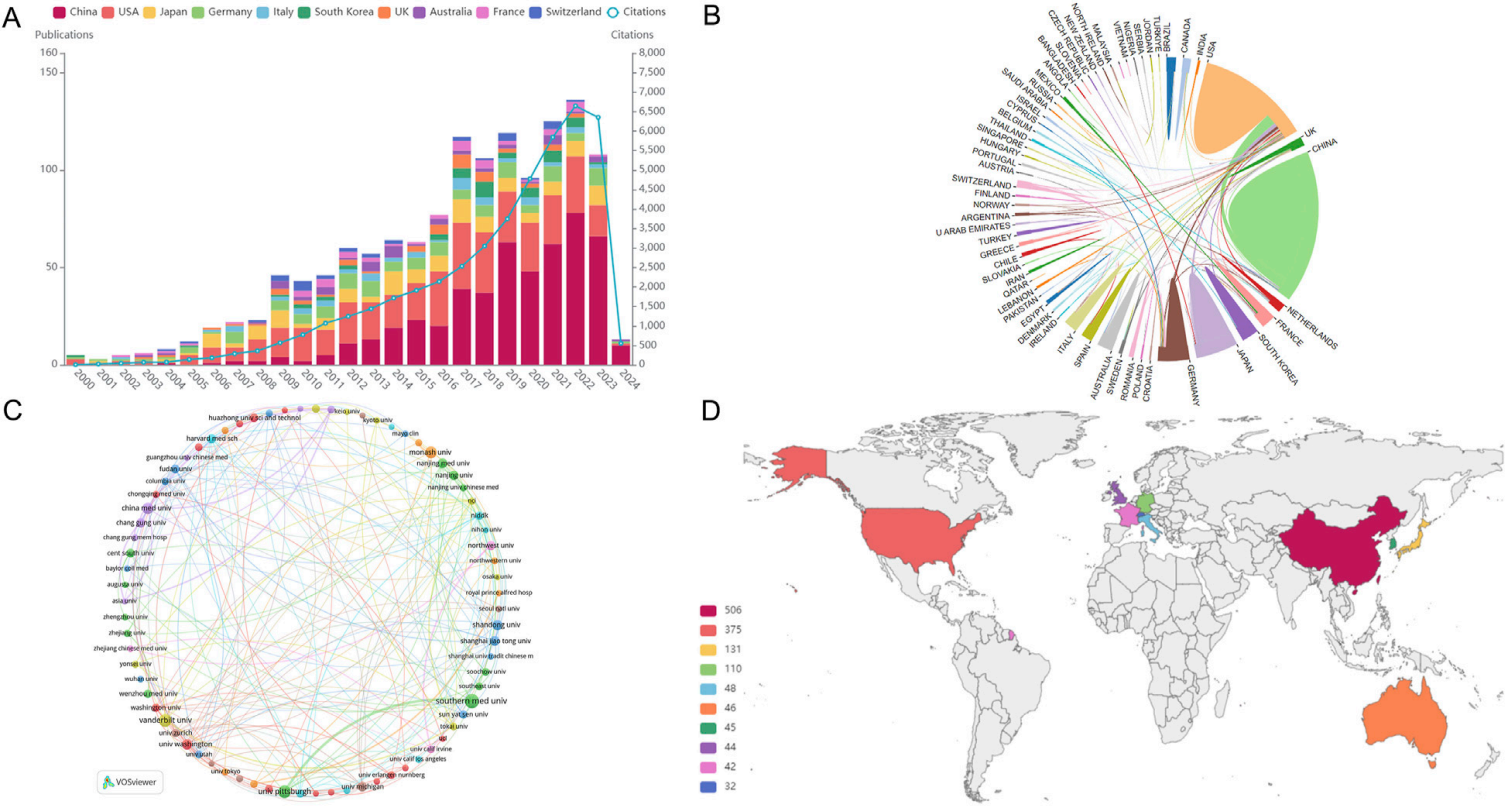
Analysis of publications, countries/regions and institutions. **(A)** Visually encapsulates the annual evolution and cumulative publication trends pertinent to this domain, spotlighting the top 10 countries at the forefront of this research endeavor. **(B)** Collaborative academic endeavors among countries/regions. **(C)** Institutional collaborative network via VOSviewer. **(D)** Global publication landscape.

Noteworthy is the trajectory of research interest for the past 24 years (Jan. 1, 2000 to Feb. 16, 2024), as delineated in Figure 1A. The examination of podocyte injury’s role in precipitating renal fibrosis has witnessed a sustained and remarkable ascent in scholarly output, indicative of the burgeoning importance of this field.

Further, these scholarly contributions boasted an average citation rate of 36.03 per paper, amassing an impressive aggregate of 43,852 citations.

### 3.2 Analysis of countries/regions and institutions

In consonance with the global distribution of research contributions, our investigation, as delineated in Figure 1D, unveils a tapestry of scholarly engagement emanating from 62 countries and regions. The comprehensive insights into the quantity of publications and their corresponding citation metrics for each country/region are meticulously cataloged in Table 1. Notably, China (506), the United States (375), Japan (131), Germany (110), Italy (48), Australia (46), Korea (45), United Kingdom (44), France (42), and Switzerland (32) emerge as the foremost contributors to this discourse. China has the highest number of publications, as exemplified in Table 1.

**TABLE 1:** Leading productive countries/regions in the relevant field.

Countries/Regions	Articles	%	Citations	Citations per paper
China	506	36.69	13,617	27.62
United States	375	27.19	21,486	57.76
Japan	131	9.50	4,052	31.41
Germany	110	7.98	5,808	53.28
Italy	48	3.48	2,543	52.98
Australia	46	3.34	2,481	52.79
Korea	45	3.26	1,124	24.43
United Kingdom	44	3.19	2,668	58
France	42	3.05	1,774	38.57
Switzerland	32	2.321	1,587	46.68

For a vivid portrayal of collaborative dynamics among nations, Figure 1B presents an interactive collaboration map, wherein the thickness of segments signifies the frequency of intercountry cooperation.

A confluence of scholarly endeavors encompassing 1,250 papers emanates from a diverse tapestry of 1,462 distinct institutions. Table 2 meticulously outlines the preeminent institutions spearheading this research with an intriguing observation. The top 10 institutions are predominantly located in the United States, China, and France, a trend that mirrors the country-level distribution of research output.

**TABLE 2:** Ranking of top 10 institutions by publications.

Affiliations	Country	Articles	%	Citations	Citations per paper
Southern Medical University China	China	41	3.4	2,128	51.9
Institut National De La Sante Et De La Recherche Medicale Inserm	France	36	3.0	1,368	38.00
Pennsylvania Commonwealth System of Higher Education	United States	35	2.9	3,637	103.91
University of California System	United States	32	2.6	2,473	77.28
U.S. Department of Veterans Affairs	United States	32	2.6	1,361	42.53
University of Pittsburgh	United States	31	2.5	3,573	115.26
National Institutes of Health	United States	30	2.5	1,575	52.5
Vanderbilt University	United States	30	2.5	1,624	54.13
Harvard University	United States	29	2.4	1,974	68.07
Veterans Health Administration	United States	28	2.3	1,229	43.89

Within institutional cooperation, as visualized in Figure 1C using VOSviewer, a more pronounced network of collaboration is discernible among United States institutions, particularly gravitating around the University of Washington, compared to Chinese institutions.

In delving into the citation analysis of institutions, Table 2 unfurls the top three institutions commanding the highest citation counts: the Pennsylvania Commonwealth System of Higher Education (3,637 citations), the University of Pittsburgh (3,573 citations), and the University of California System (2,473 citations). It is also worth noting that the research emanating from the University of Pittsburgh wielded a particularly robust impact within this scholarly discourse.

Furthermore, the University of Pittsburgh has made noteworthy contributions to renal fibrosis research, boasting the highest average citation count per paper at 115.26. The Chinese research institution Southern Medical University has the highest volume of publications in this domain, amassing a total of 41.

### 3.3 Analysis of authors and co-cited authorships

Within the ambit of our investigation, a cadre of 6,014 authors actively explored this subject matter. Table 3 provides a concise enumeration of the top 10 most prolific authors in this domain, bearing testimony to the profound contributions of these scholarly luminaries. Notably, seven authors (Liu Youhua, Zhao Yingyong, Yang Yang, Zhou Lili, Li Yan, Chen Lin, and Chen Danqian) are from China, underscoring the nation’s substantive scholarly presence in this field.

**TABLE 3:** Leading authors in the research domain.

Rank	Authors	Articles	%	Citations	Citations per paper	Co-cited authors	VOSViwer citations
1	Liu YH	34	2.79	3,411	100.32	Kriz W	237
2	Fogo AB	17	1.40	934	54.94	Liu YH	176
3	Zhao YY	16	1.31	757	47.31	Zhou LL	145
4	Yang Y	15	1.23	451	30.07	Wolf G	145
5	Zhou LL	15	1.23	1,141	76.07	Zhao YY	134
6	Li Y	14	1.15	242	17.29	Shankland SJ	127
7	Anders HJ	13	1.07	678	52.15	Kato M	125
8	Chen L	13	1.07	596	45.85	Anders HJ	124
9	Liu Y	13	1.07	277	21.31	Mundel P	121
10	Chen DQ	12	0.99	679	56.58	Zeisberg M	120

Liu Youhua, Fogo Agnes B, and Zhao Yingyong are the leading contributors, with 34, 17, and 16 publications. Furthermore, in co-cited authorships, Kriz W, Liu YH, and Zhou LL stand out as the top three, with 237, 176, and 145 citations each.

**FIGURE 2:**
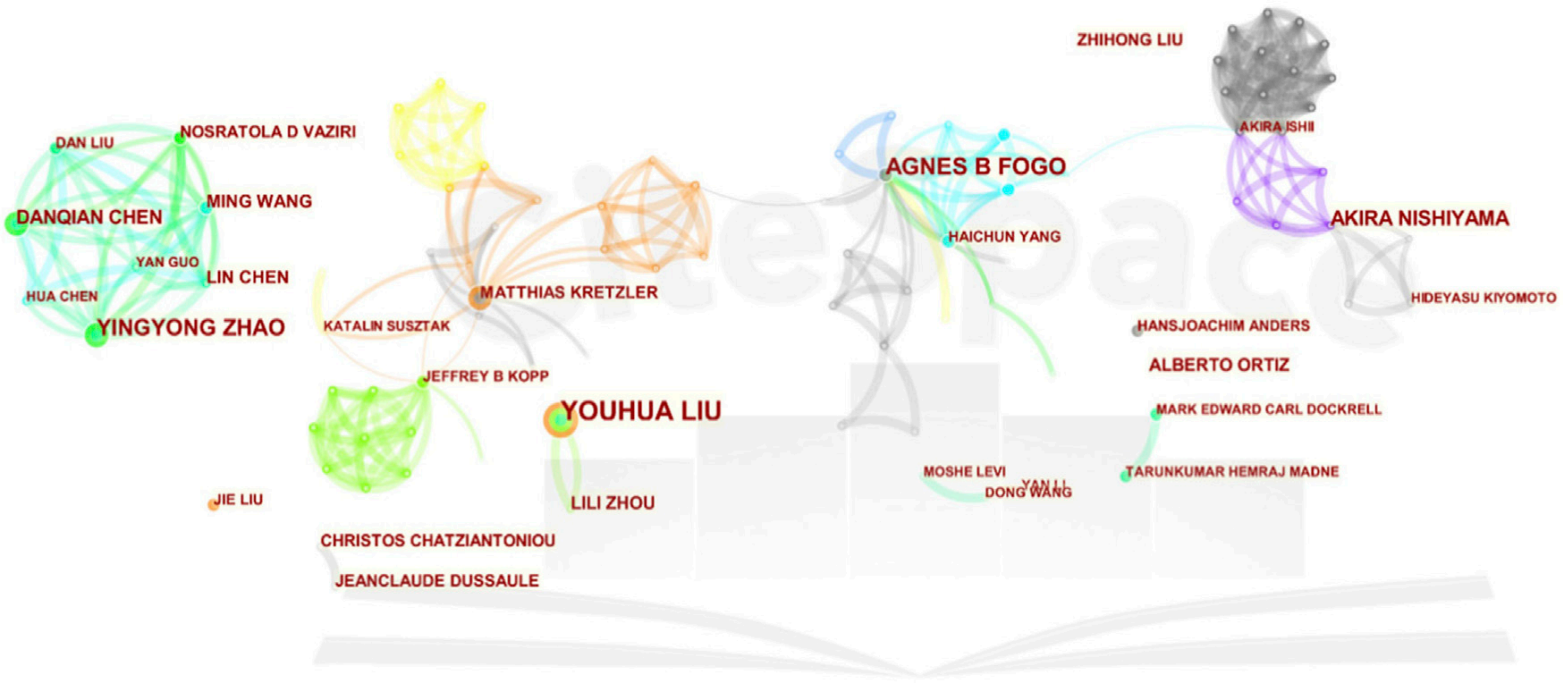
Collaborative network of authors. A visual representation delineating the cooperative networks and interactions among authors engaged in the study of this subject.

Professor Liu Youhua, a famous scholar in the State Key Laboratory of Organ Failure Research of the Southern Medical University of China, has been committed to the study of the pathological mechanism of renal fibrosis for a long time and has published more than 200 SCI papers and 2,300 influencing factors. He also serves as an assistant professor at Brown University in the United States and an associate professor at the University of Pittsburgh, demonstrating his key role and outstanding performance in this research field. Interestingly, Liu ranks in the top three among high-yield and co-cited authors, demonstrating academic strength and receiving high praise, as shown in Figure 2.

Liu research on factors related to renal fibrosis, in addition to his previous research on key factors related to epithelial-mesenchymal transition (EMT) and oxidative stress in the fibrotic microenvironment (Li et al., 2023), he believes that podocyte injury has a direct impact and leading cause on CKD and renal fibrosis. His latest research suggests that targeting Trim63 (an E3 ubiquitin ligase) may be a feasible therapeutic strategy for podocyte injury and proteinuria (Chen et al., 2023; Liu, 2010).

Fogo Agnes B from Vanderbilt University jointly participated in developing numerous guidelines; he believes that using high-dose angiotensin receptor blockers (ARBs) during urinary tract obstruction can protect podocytes and prevent glomerulosclerosis and renal fibrosis (Zhu et al., 2022).

As the academic leader of our team, Professor Zhao YY ranks third in overall research in this field. His research mainly focuses on the use of natural products for anti-fibrotic treatment. He believes that Poricoic acid A (Chen et al., 2019b) in Poria cocos serves as a regulator of TPH-1 expression (Chen et al., 2020a; Chen et al., 2019b), inhibiting renal fibrosis, stabilizing the regulatory protein of B-catenin, and mediating B-catenin transcription (Wang et al., 2018a) and natural products (including isolated compounds, crude extracts, and traditional Chinese herbal formulas) to regulate RAS (Yang et al., 2019) can inhibit the accumulation of ECM in HK-2 cells and alleviate podocyte damage and fibrosis (Chen et al., 2017).

### 3.4 Analysis of journals

A comprehensive spectrum of 366 distinct journals has been instrumental in disseminating scholarly discourse pertaining to this subject matter, with 65 journals contributing substantial articles of more than five publications each. The delineation of the top 10 journals is elucidated in Table 4, collectively encapsulating 28.51% of the scholarly contributions, totaling 347 articles. Foremost among these, Kidney International stands out, having furnished the highest number of articles (n = 65).

**TABLE 4:** Preeminent journals in the field of nephrology.

Journal	Articles	%	Citations	Citations per paper	IF	JCR	Country
Kidney International	65	5.34	4,995	76.85	19.6	Q1	United States
Journal of the American Society of Nephrology	61	5.01	6,452	105.77	13.6	Q1	United States
American Journal of Physiology-Renal Physiology	49	4.03	1,786	36.45	4.2	Q1	United States
PLoS One	37	3.04	1,032	27.89	3.7	Q2	United States
Nephrology Dialysis Transplantation	36	2.96	1,455	40.42	6.1	Q1	United Kingdom
International Journal of Molecular Sciences	30	2.47	471	15.7	5.6	Q1	United States
Frontiers in Pharmacology	25	2.05	354	14.16	5.6	Q1	SWITZERLAND
Scientific Reports	17	1.40	473	27.82	4.6	Q2	United Kingdom
Evidence-Based Complementary and Alternative Medicine	14	1.15	121	8.64	2.65	Q3	United Kingdom
American Journal of Pathology	13	1.07	1,639	126.08	6	Q1	United States

Noteworthy is the scholarly prowess of these journals, as validated by the 2022 Journal Citation Report (JCR), wherein seven of the top 10 journals garner placement in the esteemed Q1 academic ranking tier. Of particular significance, two of these journals command an Impact Factor (IF) exceeding 10, with Kidney International reigning supreme with the highest IF at 19.6.

**FIGURE 3:**
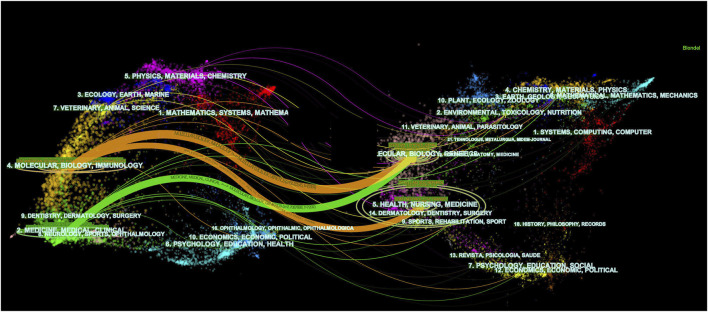
Dual-map overlap of journals in kidney repair research. A visual representation depicting the dual-mapping of journals engaged in the discourse surrounding kidney repair within the context of this research domain.

The intricate interplay of scholarly citation paths is elucidated through Figure 3, employing double-graph overlapping journals to elucidate the citation trajectory between source and cited journals. This visualization method bifurcates the citation pathway, commencing with the source journal on the left and culminating with the cited journal on the right while concurrently delineating the thematic foci of the journals. Notably, the terms “Molecular Biology and Immunology” and “Medicine, Medical, and Clinical” from cited journals evolved into one “Molecular Biology and Immunology”.

Our analysis of leading journals reveals that Kidney International, Journal of the American Society of Nephrology, and American Journal of Physiology Renal Physiology have emerged as the primary journals for kidney diseases. The remaining top ten journals are listed as follows (Table 4).

### 3.5 Analysis of co-cited references

Within the scope of this inquiry, collectively engendered the citation of a total of 29,902 references, all germane to the focal topic. Notably, Table 5 unveils the paramount co-cited references among the most cited (n = 24) and exhibiting substantial citation burst strength is the study titled “Diabetic Kidney Disease Challenges, Progress, and Possibilities,” authored by Alicic RZ (Alicic et al., 2017). This comprehensive investigation delves into the impact of intensive hyperglycemia treatment on microvascular outcomes in type 2 diabetes, in addition to offering evidence-based guidelines for the management of hypertension in adults. Furthermore, the study “Epithelial-to-mesenchymal Transition as a Potential Pathway Leading to Podocyte Dysfunction and Proteinuria,” authored by Li et al. (2008), explores the intricate pathways potentially culminating in podocyte dysfunction and proteinuria within chronic kidney diseases. This work emphasizes the role of epithelial-to-mesenchymal transition, underscoring the significance of key players such as P-cadherin, zonula occludens-1, and nephrin in podocyte functionality. The third most cited study, “The Spectrum of Podocytopathies: A Unifying View of Glomerular Diseases,” authored by RC Wiggins, presents a comprehensive review that offers a unified perspective on glomerular diseases. It underscores the pivotal role of the podocyte and underscores the importance of comprehending podocyte biology for both clinical and scientific strategies geared toward averting disease progression (Wiggins, 2007).

**TABLE 5:** Preeminent 10 co-cited references pertinent to kidney repair in this research domain.

Count	First author	Journal	Title	Year	Citations
24	Alicic RZ	Clin J Am Soc Nephro	Diabetic Kidney Disease Challenges, Progress, and Possibilities	2017	1,142
22	Li YJ	Am J Pathol	Epithelial-to-mesenchymal transition is a potential pathway leading to podocyte dysfunction and proteinuria	2008	312
20	Chen L	Chem-Biol Interact	Role of RAS/Wnt/beta-catenin axis activation in the pathogenesis of podocyte injury and tubulo-interstitial nephropathy	2017	80
20	Meng XM	Nat rev Nephrol	TGF-beta: the master regulator of fibrosis	2016	1868
20	Zhou LL	Nat Rev Nephrol	Wnt/beta-catenin signalling and podocyte dysfunction in proteinuric kidney disease	2015	164
19	Hu MS	J Cell Mol Med	LncRNA MALAT1 is dysregulated in diabetic nephropathy and involved in high glucose-induced podocyte injury via its interplay with beta-catenin	2017	160
19	Wang M	Phytomedicine	Poricoic acid ZA, a novel RAS inhibitor, attenuates tubulo-interstitial fibrosis and podocyte injury by inhibiting TGF-beta/Smad signaling pathway	2017	82
19	Wiggins RC	Kidney Int	The spectrum of podocytopathies: A unifying view of glomerular diseases	2007	606
18	Liu YH	J Am Soc Nephrol	New Insights into Epithelial-Mesenchymal Transition in Kidney Fibrosis	2010	768
18	Shankland SJ	Kidney Int	The podocyte’s response to injury: Role in proteinuria and glomerulosclerosis	2006	718

**FIGURE 4:**
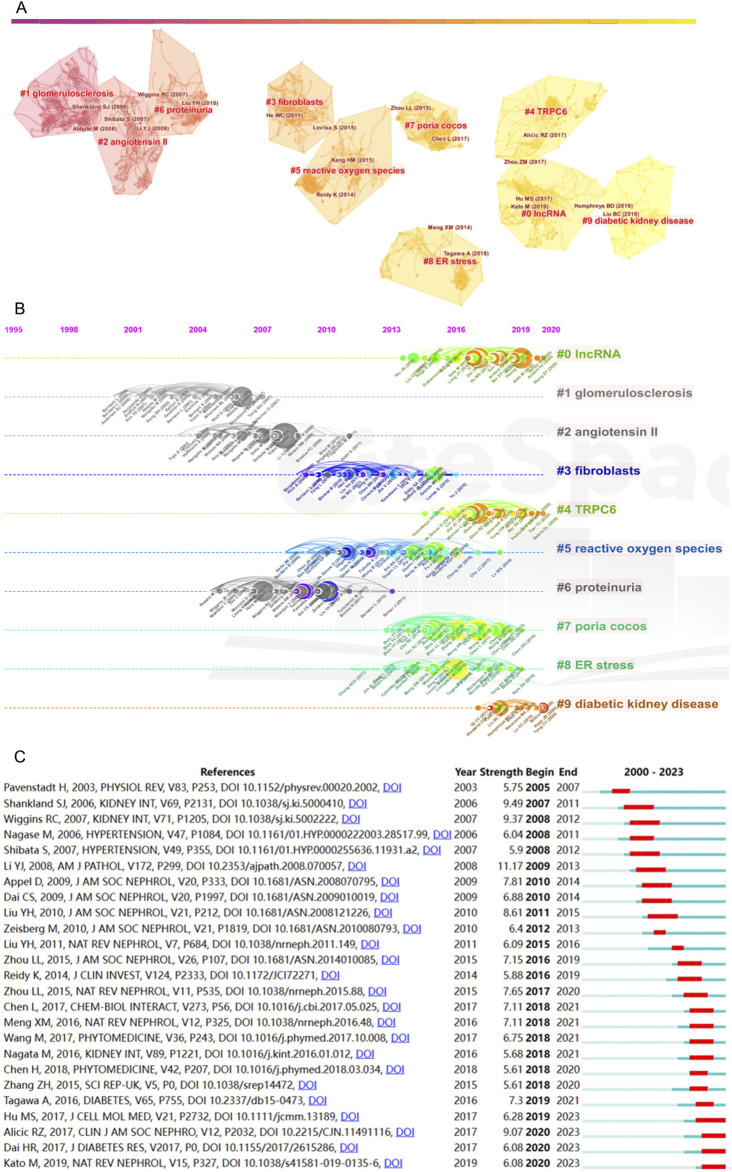
Analysis of co-cited references. **(A)** Cluster diagram of co-cited references with keywords as label source. **(B)** Temporal perspective on co-cited references through keywords. **(C)** Top 25 references with strongest citation bursts (red represents the outbreak time range).

Intricacies of the co-citations are rendered comprehensible through Figure 4A, wherein CiteSpace facilitates the visual representation of the co-citation network of references. A meticulous examination delineates the emergence of ten distinct clusters, each tethered to pertinent keywords. These clusters are identified as follows: #1 IncRNA, #2 reactive oxygen species, #3 glomerulosclerosis, #4 Poria cocos, #5 glomerular diseases, #6 fibroblasts, #7 connective tissue growth factor, #8 coagulation, #9 Wnt. Moreover, Figure 4B furnishes a temporal perspective on these co-cited references, pivotal for discerning the evolving landscape of research hotspots over time. The clusters, characterized by keyword labels, are depicted at distinct positions and hues on the timeline, delineating variances in publication chronology.

The fourth cluster, “Poria cocos”, distinguishes itself among many mechanism-related articles. Notably, this particular Chinese herbal medicine occupies the foremost position in utilizing herbal formulations for treating kidney diseases in China (Cai et al., 2011). As a clinically effective drug for kidney protection, its mechanism of action is diverse and has not yet been fully explained. In the study, the herbal medicine Poria cocos emerges as a highly cited term, indicating that Eastern herbal medicines, as significant therapeutic agents for kidney diseases, are gradually garnering attention from scholars worldwide. Poricoic acid A (PAA), derived from Poria cocos, has been identified as a modulator of TPH-1 expression, demonstrating its efficacy in attenuating renal fibrosis (Chen et al., 2020b), as well as enhancing melatonin’s inhibitory effects on the transition from AKI to CKD (Chen et al., 2019a). Additionally, Poricoic acid ZC (PZC), Poricoic acid ZD (PZD), and Poricoic acid ZE (PZE) have exhibited renin-inhibiting properties and have shown promise in safeguarding against tubulo-interstitial fibrosis (Wang et al., 2018b).

CiteSpace’s citation burst analysis, a formidable analytical tool, unfurls the references that have garnered widespread scholarly attention. Figure 4C showcases the 25 references that have exhibited the most robust citation bursts, additionally disclosing the periods during which these references sustained peak citation intensity. Four of the most recent citation bursts merit particular attention. First, the article authored by Hu MS in 2017 (Hu et al., 2017), published in the Journal of Cellular and Molecular Medicine, explores the roles of MALAT1 and β-catenin in podocyte dysfunction and kidney fibrosis, spotlighting their potential as therapeutic targets for glomerular diseases. Secondly, Alicic RZ’s work (Alicic et al., 2017) is reiterated as a citation burst. Third, the review by Dai HR in 2017 (Dai et al., 2017), featured in the Journal of Diabetes Research, dissects the mechanisms underpinning podocyte injury in DKD while illuminating novel molecular insights that hold promise as therapeutic avenues for managing this condition. Lastly, Kato M’s study in 2019 (Kato and Natarajan, 2019), published in Nature Reviews Nephrology, explores the role of epigenetic mechanisms in the occurrence and progression of DKD, explores the concept of metabolic memory in-depth, and considers that group association studies identifying epigenetic signatures of DKD may also provide information for precision medicine methods.

### 3.6 Analysis of keywords

To unravel the intricate dynamics underpinning the distribution and temporal evolution of author keywords, both a co-occurrence network map and a cluster map were employed.

**FIGURE 5:**
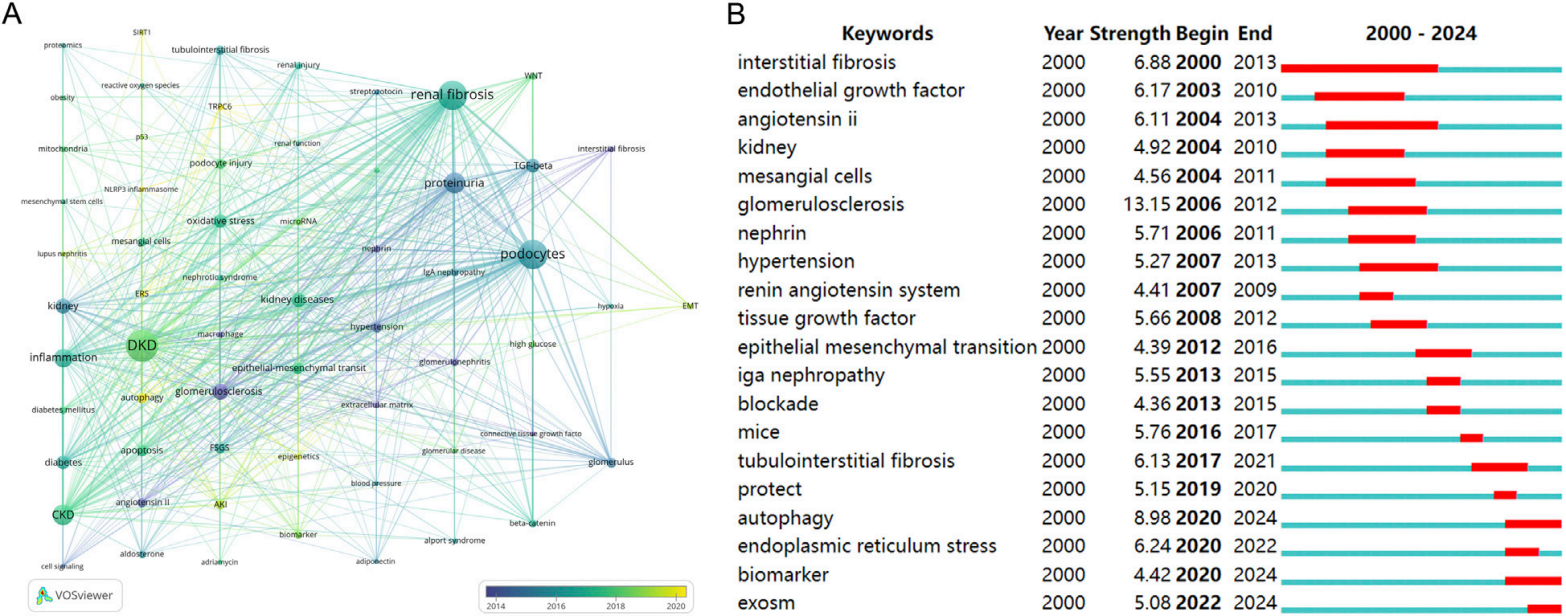
Analysis of keywords. **(A)** Cluster network of co-occurring author keywords and their temporal evolution. **(B)** Top 20 keywords with the strongest citation bursts (red represents the outbreak time range).

Figure 5A employs a distinctive color scheme to signify author keywords based on their average year of occurrence, effectively conveying the evolving temporal nuances within the research landscape. Notably, recent years have witnessed a surge in keywords such as “autophagy”, “TRPC6”, “endoplasmic reticulum stress”, “epigenetics” and “NLRP3 inflammasome”. These emergent keywords signify the current trends and areas of heightened interest.

Figure 5B, utilizing CiteSpace to analyze keyword citation bursts, reveals that “autophagy”, “biomarker” and “exosomes” are presently considered focal points of research interest and activity.

As shown in Figure 5, whether it is hot words or keywords, we can see that autophagy has significant research interests in diabetic renal fibrosis. Autophagy, a highly conserved lysosomal degradation pathway governed by signaling pathways like mTOR, AMPK, and sirtuins, assumes a pivotal role in the maintenance of cellular homeostasis across various kidney cell types, encompassing renal tubular cells, podocytes, mesangial cells, and glomerular endothelial cells (Tang et al., 2020). Dysregulation of autophagy has been implicated in the pathogenesis of diverse renal pathologies. Zhao et al. (2019), for instance, conducted an in-depth analysis of the pathological implications and regulatory mechanisms of autophagy in renal fibrosis and associated kidney ailments, spanning both glomerular and tubulointerstitial compartments. Moreover, Liang et al. (2022) reported findings indicating that Qidan Dihuang decoction effectively mitigates diabetic renal injuries and fibrosis by modulating the PERK-eIF2 alpha-ATF4 pathway and promoting autophagy in DKD.

Both paths can be seen in Figures 5A, B. Endoplasmic reticulum emergency has significant research interest in diabetic renal fibrosis and podocytes. The delicate balance between the cytoprotective and cytotoxic effects of endoplasmic reticulum stress (ERS) activation is a subject of paramount significance. Pharmacological interventions that restore ERS to homeostatic levels hold immense therapeutic promise in preventing or arresting the progression of kidney-related pathologies (Cybulsky, 2017). Recent investigations have notably underscored the pivotal role ERS plays in both acute and chronic kidney diseases, particularly in renal fibrosis (Ke et al., 2017). ERS modulation emerges as a promising therapeutic avenue, potentially influencing renal fibrosis through a multitude of signaling pathways, culminating in podocyte injury. The seminal work by Bai XY further advances our understanding by elucidating the role of long intergenic non-coding RNA (LINC01619) in functioning as a competing endogenous RNA, orchestrating miR-27a/FOXO1-mediated ERS modulation and subsequent podocyte injury, as observed in DKD (Bai et al., 2018).

Transient receptor potential cation channel 6 (TRPC6) is a glomerular cleft diaphragm-associated channel required for normal renal function. DKD is associated with impaired podocyte autophagy and subsequent podocyte damage. However, the regulation of podocyte autophagy is unique and is related to the loss of calcium regulation homeostasis, leading to podocyte damage (Ilatovskaya et al., 2018). Diabetes increases the expression of TRPC6 in podocytes *in vivo* while reducing podocyte autophagy flux (Staruschenko et al., 2019) TRPC6 gene knockout can reduce the progression of DKD (Salemkour et al., 2023; Ma et al., 2019). Kim et al. (2018) discovery elucidates a causal link between mutations within the classic TRPC6 and manifestations of rare familial cases of focal and segmental glomerulosclerosis (FSGS). Substantial research has been diligently conducted, particularly on comprehending the intricate regulatory mechanisms governing TRPC6 channels, particularly within the context of podocyte function (Dryer et al., 2019). However, some scholars believe that fibrosis is not caused by the influx or outflow of calcium ions in podocytes. Electron microscopy shows that defective mutations in this gene do not cause FSGS changes. Rather than through TRPC6 increasing rather than decreasing calcium influx for podocyte death (Batool et al., 2023).

Epigenetics, an encompassing term for the study of heritable changes in gene function that are not rooted in alterations to the DNA sequence itself, has assumed pivotal importance in the context of renal fibrosis associated with podocyte dysfunction. These epigenetic modifications exert regulatory control over various genes implicated in the pathogenesis of renal diseases, including fibrosis and inflammation (Kato and Natarajan, 2019). Notably, recent research has unveiled the intricate interplay between epigenetic machinery and microRNA (miRNA) expression patterns, particularly in various disorders, including DKD (Sankrityayan et al., 2019). Furthermore, Rai et al.’s study underscores the significance of the epigenetic regulator FATp300 in fibrogenesis, identifying it as a prospective therapeutic target for mitigating pathological matrix remodeling and associated pathologies. Additionally, L002 emerges as a novel therapeutic candidate, potentially ameliorating hypertension-induced cardio-renal fibrosis while impeding pro-fibrogenic responses in fibroblasts, podocytes, and mesangial cells (Rai et al., 2017).

The kidney, characterized by heightened energy requirements, is replete with an abundance of mitochondria. Quadri’s investigative endeavors have underscored the pivotal involvement of mitochondrial dysfunction in the physiological progression of renal fibrosis (Quadri et al., 2019). The perturbation of mitochondria culminates in the release of dangerous molecules, including reactive oxygen species, DNA, and cardiolipin, which subsequently trigger the NLR family pyrin domain containing 3 (NLRP3) inflammasome activation and the upregulation of interleukin-18 (IL-18) and interleukin-1 beta (IL-1β) (Szeto et al., 2017). Pharmacological inhibition of the NLRP3 inflammasome has proven effective in ameliorating renal injury across diverse animal models (Chang et al., 2014). Moreover, the activation of purinergic 2X_7_ receptors (P2X_7_R) in conjunction with the NLRP3 inflammasome has been identified as a significant contributor to renal inflammation and injury within the context of metabolic syndrome-related renal ailments. Notably, studies involving mice devoid of P2X_7_R have demonstrated reduced inflammation, diminished oxidative stress, and attenuated fibrosis, underscoring the therapeutic potential of these molecular targets in mitigating renal inflammation and injury associated with type 2 diabetes and metabolic syndrome (Solini et al., 2013; Li et al., 2022).

Furthermore, Figure 5B discerns research hotspots by identifying keywords that exhibit robust citation bursts. Keywords such as “autophagy”, “biomarker” and “exosomes” continue to experience sustained citation bursts, suggesting their potential to evolve into enduring research focal points.

## 4 Discussion

### 4.1 The trend analysis and contributions of countries over two-decade

From the development history of DKD, in the 1950s, the United States and Europe began to have the first record of *in vivo* renal biopsy technology and recorded the glomerular fibrosis of diabetic patients (Cameron, 2006). The subspace structure of podocytes, first described in 2005, was studied by Neal et al. (2005) on how it affects the microenvironment of podocytes, as well as filtration and lateral shear stress. The importance of podocytes as target cells in the pathogenesis of kidney diseases is self-evident. The main characteristics of podocytes as molecular sieves are their regularly intersecting foot processes and bridge structures used for kidney filtration. The dedifferentiation response factor of immortalized human podocytes in response to TGF-β and other TGF-dependent stimuli leads to dedifferentiation leading to the disappearance of foot processes, hypertrophic morphology and increased formation of intercellular tight junctions, which leads to the formation of fibrosis (Herman-Edelstein et al., 2011). Current anatomical studies indicate that approximately 60% of the glomerular filtration surface is covered by this space. Agnes B Fogo believes that podocyte loss has a severe impact on renal fibrosis (Fogo, 2021). From early this century, a notable escalation in annual publications about podocyte-associated diabetes and renal fibrosis is evident, which has a substantial spike in publications occurred from 2017 to 2023. It is extremely displaying that the research on renal fiber and chronic kidney disease caused by diabetes is still under in-depth exploration as a prevailing subject of research inquiry on podocytes. This research shows that the United States, China, and Japan are the top three countries with the most significant publications. The research field of diabetes and nephrology in China has made rapid progress, especially in the past 10 years. Seven of the ten authors form an essential team from China, indicating substantial academic influence in this field.

### 4.2 PAA as an effective complementary therapy for DKD and renal fibrosis

As to new therapies, Professor Zhao Yingyong mainly studied the protective mechanism of natural molecular compounds of Poria cocos on the kidneys and its anti-renal fibrosis effect (Chen et al., 2020b; Chen et al., 2019a; Wang et al., 2018b; Wang et al., 2023). In preliminary research, he has identified a small molecular component, PAA. It potentially holds considerable potential in mitigating renal damage caused by DKD, such as the fibrotic differentiation of podocytes and renal epithelial cells into fibroblasts, as well as in inhibiting renal fibrosis. He is also the third globally cited author.

PAA is a component isolated from the traditional Chinese medicine Poria cocos, which has hypoglycemic and anti-fibrotic effects. As a clinically effective renal protective drug, its mechanism of action is diverse and not yet fully explained. It has been confirmed that is related to improving podocytes. In fact, China, with a history of more than 5,000 years, has believed that diabetes is closely related to the kidney since ancient times, and Traditional Chinese Medicine (TCM) believes that the kidney is the key. Based on the academic theories of traditional Chinese medicine (as described in Classic medical books, Ling Shu (Unschuld, 2016), published for thousands of years, covering kidney Qi and blood energy and ion exchange), it provided more theoretical background for treatment. At the beginning of the third century AD, Zhang Zhongjing, a famous physician of the Eastern Han Dynasty, wrote in his book “Synopsis of Prescriptions of the Golden Chamber” (Zhang and Luo, 1987), “Men with kidney diseases may experience abnormal urination, sweetness in urine and frequent urination. At this time, it is necessary to take Shenqi pills.” A solution to this problem was proposed, and the famous formula “Bawei Shenqi Pills” (Liuwei Dihuang Pills, containing Poria cocos) was given in the “Xiao Ke Disease” chapter, which has been used for thousands of years.

At present, classic Chinese formulas have excellent vitality, which is also supported by co-citation literature analysis. In addition, PAA significantly reduced blood glucose and urinary protein levels in DKD mice and inhibited renal fibrosis. PAA can promote mitochondrial autophagy by downregulating FUNDC1, thereby having a beneficial effect on podocyte damage in DKD renal fibrosis. This indicates that traditional Chinese medicine molecular compounds, as important therapeutic drugs for kidney diseases, are receiving attention from scholars worldwide for their therapeutic effects.

### 4.3 Emerging prospective drugs and mechanism research hotspots

DKD development is characterized by intricate cellular and molecular interactions involving podocytes, glomerular endothelial cells, and mesangial cells. These core glomerular components are vital in their reciprocal impacts and pathological changes. Communication among these cells occurs via diverse signaling cascades, including those governed by angiopoietins, VEGF, TGF-β, Krüppel-like factors, RARRES1 (Hu et al., 2024), and extracellular vesicles, fostering essential intercellular communication for preserving glomerular filtration barrier function and renal homeostasis. Oxidative stress is a driving force behind podocyte hypertrophy, with elevated TGF-β1, Ang II, and mTORC1 expression contributing to this phenomenon under hyperglycemic conditions. Podocyte epithelial-to-mesenchymal transition is implicated in proteinuria onset, with Wnt/β-catenin, SDF-1α, and PI3K/AKT pathways triggering this process. Apoptosis, via both extrinsic and intrinsic mitochondrial pathways, contributes to proteinuria and glomerulosclerosis in DKD. Additionally, a newly developed drug treatments, Finerenone, the non-steroidal mineralocorticoid receptor antagonist, has demonstrated significant promise in the treatment of DKD (Fujii and Shibata, 2023). Research findings suggest that it improves mitochondrial dysfunction in renal tubular cells, mitigates inflammation and fibrosis, reduces proteinuria, and retards the progression of CKD (Barrera-Chimal et al., 2022).

The development of renal fibrosis in patients with DKD is closely tied to abnormalities in multiple signaling pathways, with the overactivation of the TGF-β pathway emerging as a principal mechanism contributing to fibrosis. Researchers are actively investigating means to suppress this pathway, along with the expression and activity of other pertinent inflammatory factors, in an effort to potentially retard or reverse the fibrotic process (Liu et al., 2024). During EMT, podocytes should lose their epithelial polarity, intercellular junctions would be altered, and the actin cytoskeleton will be rearranged (Shankland, 2006). After stimulation with TGF-B1, podocytes lose renin and ZO-1 expression (Sun et al., 2023). As important slit diaphragm proteins, loss of expression of renin and ZO-1 is detrimental to podocyte function (Zhao et al., 2022). Podocyte autophagy, a type II programmed death, initiation is crucial in the progression of podocyte loss and massive proteinuria.

Research on gene regulation and epigenetics, the research team led by Professors Zhang DS and Dong Z have elucidated the role of the PRDM16 gene during the relatively inconspicuous stage of tubulointerstitial fibrosis in the early phase of DKD (Wang et al., 2017). Their findings show that the introduction of a viral vector encoding PRDM16 or the use of the small-molecule compound Formononetin can attenuate renal fibrosis in DKD.

GLP-1 receptor agonists (GLP-1RAs) (Sourris et al., 2024) have been found to exhibit not only potent glycemic-lowering effects, but also marked reno protective properties. Multiple clinical trials, including the LEADER study, have provided compelling evidence that these agents confer noticeable renal benefits in individuals with DKD.

Scientists are currently exploring integrated therapeutic interventions targeting multiple key aspects of the fibrosis cascade, encompassing but not restricted to the inhibition of fibroblast activation, modulation of ECM homeostasis, counteracting oxidative stress, and improving microcirculatory dysfunction (Cotas et al., 2024). Moreover, TCM characterized by their multitarget nature, hold great promise in treating DKD, making their effects highly anticipated in disease management (Wang et al., 2024).

In summary, a deeper understanding and exploration of these mechanisms hold promise for the development of novel therapeutic strategies aimed at protecting podocytes in DKD and treating renal fibrosis.

### 4.4 Will HIM diagnosis and treatment coming?

A multidisciplinary development trend is evident within the top 10 journals dedicated to this subject. The critical researches span various domains, including Nephrology, Cell research, Pharmacology and Molecular Biology, which illustrate the multifaceted and complex research in this field.

Clinicians may be familiar with the fact that some patients with diabetes have high blood sugar, which will cause their kidneys to suffer multiple blows for a long time, promote renal fibrosis, and then it is lead to the decline of renal function until that progresses to renal failure and even renal replacement therapy. This is a common clinical disease transformation and bad outcome. Here, the treatment and diagnosis process covers the responsibilities of the Endocrinology, Nephrology, and Urology departments, etc. However, is this really beneficial for patients?

In response to this dilemma, it is worth that trying the combination of HIM. Chinese academician Fan Daiming. Has already proposed the concept of Holistic Integrative Medicine (Fan, 2017), and he has proposed that the growth of medical knowledge, drug varieties, and technological advancements, coupled with rising health demands, needs a more comprehensive approach. Moreover, the medicine revolution offers opportunities for HIM, such as “Doctor Cloud”, an intelligent platform that expands doctors’ knowledge, accumulates experience, and provides precise patient services. Fogo AB, another expert in this field, his latest article on JAMA aims to demonstrate that marking should done by experts, allowing the accuracy of computer diagnostics to meet the highest professional standards. The combination of AI and medicine is worth thinking in depth, using the field of renal pathology as an example to illustrate its development, emphasizing that this field is only a model for other medical fields (Fogo et al., 2024). Experts have been discussing this point of view. However, it seems that the revolution in computing is integrating medicine. By leveraging AI and data bank, one platform forms a “medical brain”, allowing doctors nationwide to seek solutions for challenging clinical cases in real-time just around the corner. Internet technologies enable personalized integration plans tailored to individual patient needs, preliminary describing the future of HIM medical development.

Furthermore, HIM medicine model is used to provide personalized intervention to the research subjects. To Integrated medicine model: Endocrinologists, cardiologists, general medicine physicians, traditional Chinese medicine practitioners, nutritionists, psychological counselors, exercise managers, pharmacists, health managers, and basic medical professors jointly participate in on-site education and discussions. To develop a comprehensive intervention plan participate in one-to-one personalized management offline and online, and implement personalized anti-diabetes treatment from seven dimensions: Western medicine prevention and treatment, psychological counseling, nutritional intervention, exercise intervention, traditional Chinese medicine conditioning, health monitoring, and rehabilitation guidance plan. A team of senior professional title experts and excellent professional and technical qualifications from high-level hospitals, medical universities, and diabetes-related research institutions conduct offline and online on-site health education and discussion venues and clinics, and try to integrate them into a more complete system as much as possible. The environment is bound to be conducive to the development of doctors, patients and related research as a whole. Standing on the shoulders of many giants and rethinking the course and related progress of this disease is a feasible treatment strategy worthy of further research and thinking.

## 5 Conclusion

This study applied bibliometric to visualize the interplay between podocytes, DKD, and renal fibrosis from 2000 to 2024. Diabetes pose an inevitable challenge for society’s development. Despite a steady growth on podocytes’ role in DKD and renal fibrosis, the global scientific community has yet to fully elucidate their mechanisms and functions. This bibliometric analysis proposed key terms and novel therapies, including molecule compounds derived from TCM, which may offer valuable insights into the elusive mechanisms of podocytes. These findings deserve significant attention from nephrologists and related researchers.

## Data Availability

The original contributions presented in the study are included in the article/supplementary material, further inquiries can be directed to the corresponding authors.
